# The Value of Implicitness: An Empirical Ethics Analysis of Indonesian Everyday Family Involvement in a Palliative Care Setting

**DOI:** 10.1007/s41649-025-00360-6

**Published:** 2025-04-16

**Authors:** Raditya Bagas Wicaksono, Amalia Muhaimin, Dick L. Willems, Jeannette Pols

**Affiliations:** 1 Department of Ethics, Law, and Humanities, Amsterdam UMC, University of Amsterdam, Amsterdam, The Netherlands; 2 Department of Bioethics and Humanities, Faculty of Medicine, Universitas Jenderal Soedirman, Purwokerto, Indonesia; 3 Amsterdam Public Health Research Institute, Amsterdam UMC, Amsterdam, The Netherlands; 4 Department of Anthropology, University of Amsterdam, Amsterdam, The Netherlands

**Keywords:** Palliative care, Family, Implicitness, Empirical ethics, Ethnography, Javanese, Indonesia

## Abstract

The lack of professional support for patients needing palliative care in Indonesia leads to a heavier reliance on family members for care. However, family tensions often arise from unmet expectations about support from other family members. This study explores implicitness, which we describe as the use of indirect or unspoken methods to communicate messages. We argue that the value of implicitness strongly influences the communication of these expectations and affects family caregiving dynamics. This paper aims to discuss the hidden expectations shaped by implicitness, what makes it an important value in family care, its ethical implications, and strategies for resolving the problems that arise from it. We conducted ethnographic fieldwork in Banyumas, Indonesia, involving in-depth interviews, home observations, and focus group discussions with patients, families, and health professionals. Data were analyzed through an empirical ethics approach. Our findings indicate that implicitness shapes family expectations regarding involvement of other family members in daily caring activities and financial support. Implicitness serves as an important value as it seeks to preserve sincerity and also maintain harmony, by not directly asking for help. However, implicitness can also lead to underlying family tensions that hinder proper care and reduce the well-being of patients and families. Families were able to resolve problems by accepting difficult circumstances or sharing them with health professionals. We suggest that health professionals should acknowledge the role of implicitness and use active listening skills to identify potential problems. If appropriate, they could help persuade family members to be more actively involved in caregiving through indirect communication. By doing so, they may enhance the well-being of both patients and families without needing every expectation to be directly and explicitly articulated.

## Introduction

In Indonesian society, families are expected to provide care for patients needing palliative care out of spiritual and normative values (Kristanti et al. 2019), without this being officially documented in the medical guidelines (Park et al. 2022). Due to a lack of professional palliative care support, particularly in rural areas of Indonesia (Martina et al. 2023), there is a heavy reliance on family members to provide care for these patients (Effendy et al. 2015; Kristanti et al. 2017; Wicaksono et al. 2024b). Although close family caregivers do provide most of the hands-on care (Kristanti et al. 2017), they cannot – and are not expected to – manage by themselves. Other family members’ direct support is still necessary and expected (Grant 2010). In an earlier article (Wicaksono et al. 2024b) we show how patients and families in Java, Indonesia, utilized intricate care networks from their family and surrounding community to support the patient.

Local culture and communication style can influence the way families organize palliative care and affect patients’ and families’ well-being. In various cultural contexts, high levels of contextualized communication are used (Everdingen and Waarts 2003), meaning people refrain from directly speaking their opinions and use communication symbols or codes instead (Endraswara 2018). Thus, actual opinions, preferences or intentions often remain implicit. In Javanese culture, people are known for their highly contextualized communication (Meyer 2016). The value of *urmat* (respect) in Javanese culture might also play a role in shaping implicitness. Being humble and refraining from showing arrogance or superiority of views are widely seen as virtues in Java (Andriyanto et al. 2022). In this case, people might refrain from explicitly requesting something from their wider family due to the value of respect. Maintaining peace and harmony is another important norm within Javanese communities. Javanese people therefore often do not express their views directly, or make compromises when faced with differences in opinion, to prevent escalation or conflict. It might be the case that when people remain implicit in their wishes, needs, and expectations, they act to preserve harmonious interaction within their family (Andriyanto et al. 2022).

From our ethnographic fieldwork in Java, here we further investigate family expectations regarding how to provide good care. Within these expectations, an implicitness in terms of what is expected or wanted, without being directly stated, plays a big role in influencing how patient care needs are communicated between family members. The term of implicitness *(tersirat* in Bahasa Indonesia*)* refers to the quality or condition of being implicit, meaning it is implied or suggested but not directly expressed or clearly formulated (Oxford English Dictionary 2023). In this study, we focus on the implicit communication regarding the involvement of family members in providing care for patients requiring palliative care. Yus (1999) states that implicit communication involves conveying messages indirectly, requiring the listener to interpret the meaning. This method is often used to communicate multiple layers of information, provide subtle reasons, and manage social interactions such as politeness.

Instances of implicitness identified in a previous Javanese study include the use of indirect language for commands, requests, or refusals (Atmawati et al. 2024). Another example includes conveying the intended message to someone other than the original recipient. In Javanese culture, implicit or indirect communication is used to show respect, maintain politeness, and avoid conflicts (Atmawati et al. 2024). Research suggests that pseudo-directive speeches are used to convey different meanings (Rahardi et al. 2023).

The theory of informational social influence popularized by Deutsch and Gerard (1955) explains that people are influenced by a majority when they are uncertain. This influence could extend to become the unspoken rules and implicit social roles which are enforced by group norms, leading to implicit expectations (Heinzen and Goodfriend 2021). In this case, the expectations are then translated into normative stereotypes about how families should behave in relation to caring for ill family members and enforced by the community. Therefore, implicit norms and expectations remain subtle, unspoken, unwritten, informal rules. This is contrary to explicit expectations, which are clearly stated through requests or demands.

Although the family is culturally expected to provide care for patients needing palliative care, it remains unclear from the current literature what expectations family members hold in palliative care situations, where these expectations come from, and how implicitness influences these expectations. Therefore, this paper aims to answer the following research questions:

1. What expectations are shaped in implicit ways?
2. Is implicitness a value in family care, and if so, how?
3. What are the ethical implications of this value?
4. How might tensions that arise from implicitness be resolved?

In this paper we examine what makes implicitness a value and what ethical implications it brings. We argue that it is crucial to critically explore and understand what makes implicitness a value, because it contains other values that people think are important, and because this value is not just relevant in Java, but probably other settings where Western ideas of 'making things explicit’ do not always work in practice. Our analysis aims to contribute to more culturally sensitive and contextually appropriate palliative care, and to global bioethics, by being responsive to the value of implicitness as a part of cultural diversity (Tosam 2020).

## Methods

We conducted ethnographic research that explored concepts, practices, and experiences of home palliative care in Banyumas, Central Java, Indonesia. We used ethnographic methods because these would enable us to closely observe activities and relations between the actors involved in care practices (Pols 2015; Strudwick 2021). Ethnography allowed us to uncover the notion of implicitness as a value through prolonged exposure during home observations and in-depth interviews. We organized focus group discussions to help us understand the dynamics among health professionals in responding to the value of implicitness. The first author conducted ethnographic fieldwork.

### Recruitment and Sampling

The first phase of ethnographic fieldwork was between June 2022 and January 2023. Purposive sampling was used to select the participants. We approached patients needing palliative care and their families based on the recommendations of local health professionals. We also contacted health professionals and institutional representatives who were involved in caring for these patients. We recruited the participants after confirming their eligibility and obtaining informed consent. Participants were deemed eligible for this study based on the criteria in Table 1.

**Table 1:** Inclusion and exclusion criteria for study participants (Wicaksono et al. 2024b)

Participant type	Inclusion criteria	Exclusion criteria
Patient	Diagnosed with life-threatening or life-limiting disease Receiving home-based care from a healthcare facility	Patients with clinically unstable conditions and having trouble in comprehending and communicating clearly. They may be included for observation but excluded from in-depth interview
Family member	Being the spouse, child, parent, or relative responsible for the patient’s care Already providing care for the patient for at least one month Living with the patient or providing daily care to the patient	Having trouble in comprehending and communicating clearly
Healthcare worker	General practitioner, medical specialist, nurse, midwife, or other healthcare professionals involved in the care of patients with life-threatening or life-limiting diseases	
Institutional representative	From the regional office of the national health insurance (BPJS) organization, regional health department, and a health philanthropic organization	

We conducted the second phase of ethnographic fieldwork from June to August 2024. We contacted some patients, families, and health professionals who had participated in the first phase. We also contacted additional health professionals from hospitals and primary health care facilities who were involved in caring for palliative patients and invited them to participate in focus group discussions.

### Data Collection

In the first phase, we conducted in-depth interviews with patients (n = 8), family caregivers (n = 10), general physicians (n = 6), medical specialists (n = 7), nurses (n = 10), other healthcare workers (n = 5), and institutional representatives (n = 3). The in-depth interviews, ranging from 17 to 88 min, explored the conceptualization of good care and family involvement in caring for palliative patients. Home observations were conducted in 12 patient-family units to observe how care was provided at home as well as to examine verbal and non-verbal communication and family dynamics in practice.

In the second phase, we conducted additional in-depth interviews with patients (n = 3) and family caregivers (n = 4) to confirm and clarify our initial analysis. We also conducted two focus group discussions, ranging from 120 to 150 min, to discuss how health professionals experienced and responded to family involvement in caring for palliative patients. The first focus group discussion involved health professionals from hospitals (n = 9), and the second involved those from primary health care facilities (n = 9).

The first author recorded in-depth interviews and focus group discussions and took fieldwork notes during home observations. All data were in Bahasa Indonesia.

### Data Analysis

The verbatim transcriptions of the interviews and focus group discussions, together with fieldwork notes, were included in a single MAXQDA (qualitative data analysis software) file project. All changes and reflections during data analysis were recorded in the file to ensure a transparent audit trail. Data obtained from the fieldwork were analyzed with an empirical ethics approach, an approach to analyze the differences and conflicting notions of what constitutes good care in practice. It could also help us to understand how people shape the concept of goodness in everyday life, particularly concerning palliative care, by including the objects, activities, and words used by people within care practices (Pols 2015, 2024).

The first author conducted initial data analysis using MAXQDA and discussed the translated initial categories and themes with all authors, along with some translated quotes from the data. Biweekly meetings with all authors were held to discuss, reflect, revise, and conceptualize the themes of the study. We report the ethnographic data with pseudonyms to preserve the anonymity of our participants and provide reference codes for the type of participant. Examples of these codes are F01 (family caregiver number 1), P02 (second patient), FGD 2 (second focus group discussion), and FW06 (sixth fieldwork notes).

We discuss the themes from the study pertaining to this paper in the next sections. The first section explores the participants’ hidden expectations about how to provide good family care, shaped by the value of implicitness. In this section, we also examine how implicitness manifests in the organization of family care. The second section analyzes what makes implicitness an important value in family care. The third section focuses on the ethical implications of this value, describing the underlying tensions and challenges caused by implicitness. The last section addresses strategies used by patients, family members, and health professionals for resolving problems arising from this value.

## Results

### Hidden Expectations Shaped in Implicit Ways

From the ethnographic data we found that implicitness influences expectations of how to provide good palliative care. Patients, family members, and health professionals had their own ideas of how a family should be involved in care. They had pre-existing ideas or conceptualizations of what constitutes good care and who provides or should provide that care. These ideas and concepts were also acknowledged by health professionals. In our findings, most of the expectations indeed remained unspoken, preventing them from becoming a request or demand.

There were variations in the degree or extent of explicit or implicit communication styles among family members, depending on each family’s communication style and cohesiveness, which we found was partly related to socio-economic status. For instance, one middle-income family in our sample that had previously lived in Jakarta and now resided near the center of Banyumas had more explicit communication between family members. Meanwhile, other families living in rural areas with lower incomes tended to have more implicit and unspoken communication. Despite differences in communication styles between families, we argue that to some extent there is always an element of implicitness in family exchanges.

We found two common unspoken expectations among families of palliative patients: that family members should be sincerely involved in daily caring activities and take the initiative in providing financial support.

#### Being Sincerely Involved in Daily Caring Activities

Families felt it was ideal when there was a daily, constant presence of a family member who could care for the patient, either at home or during appointments in health care facilities. The primary family caregivers are ‘on duty’ for almost 24 h since they are living together with the patients. The duties range from caring for the patient’s hygiene, nutrition, medication, and wounds. In a previous article (Wicaksono et al. 2024b) we provide more analysis of how patients’ family caregivers in Banyumas organize care at home for the patient, such as making an in-bed urine pipe installation and using traditional medicine.

Suyono, the husband of an elderly woman with chronic diabetes and a foot ulcer told us that caregiving is considered an “obligation”:

*Interviewer: How do you provide hygiene care for your wife?*

*Suyono: I wipe her body twice a day, in the morning and in the afternoon here on the bed. I also change her clothes. Yes, I could not go anywhere, I have to be with her. (laughing). This is an obligation.*

*Interviewer: What kind of obligation is that, sir?*

*Suyono: It is an obligation for the husband, when the wife is sick, then he has to take care of her, in every single way. (FP03)*

Here, the husband emphasized that it is an obligation for the spouse to provide care for the ill patient and be there for the patient. His wife never asked him to provide care and to be there for her, but he did it on his own initiative. This initiative-taking is a component of the notion of sincerity that we encountered in the fieldwork. Since providing care is physically and mentally demanding for the primary family caregiver, other family members are also expected to provide direct support, to take over the caregiving duty, even if temporarily. For instance, Suyono's daughter helped with washing the patient's hair and cooking food for the family. The sincerity here extends from being physically present to whole-heartedly catering to the patient and primary family caregiver’s physical and emotional needs, *without expecting a return*.

The notion and practice of sincerity was illustrated through the presence of the patient's spouse or the primary family caregiver's spouse. Their presence is important not only to help with physical caregiving activities, but also to give sincere support. They could understand each other’s capacity to do physical caring activities, even without a clear guideline on *who* will do *what*. This intuitive understanding came from long-term interactions between spouses or other family members, often requiring tinkering with caregiving roles and activities. In return, the patient would feel grateful and then pray to God for the goodness and well-being of the family members. This is shown in Mawar’s story, a patient with breast cancer:

*Mawar: My husband washes my hair and body every day, with warm water. He is very determined. He may look ugly and old, but Alhamdulillah (praise Allah), early in the morning he cooks rice and food and asks me what I want to eat. He washes our clothes and works in the rice field. [...] I pray, “Dear Allah, give me health and long life; give mercy to my husband.” I feel sorry for my husband, he must be tired, and no one helped him. (P10-1)*

This quote shows Mawar’s gratefulness for her husband’s sincere caregiving. He took care of her in every detail. Her husband did not refrain from showing his affection by crying and worrying about her condition. This led to a growing cohesion within Mawar’s family.

Not only are family caregivers expected by patients and health professionals to care for patients at home, they are also expected to accompany the patient for appointments at public health centers (*Puskesmas*) or hospitals. Health professionals expect families to accompany the patient during hospitalization. Some family caregivers showed their sincerity and full attention by accompanying patients to routine medical check-ups or appointments. However, there were other family caregivers who were not able to do so due to busyness at work, or who did not realize the importance of family involvement in these appointments. This meant that in some instances, patients were not able to attend routine check-ups because there was no one to escort them to the *Puskesmas*. At other times, family members just dropped the patient in front of the building and waited outside. Health professionals felt sorry for the patients, as the patients needed more guidance to follow the procedures inside the building, and because health professionals expected family caregivers to be there by the patient's side so as to also listen and subsequently better care for them.

Patients’ expectations that certain family members should be present could sometimes not be expressed explicitly, due to their fear of being a burden (*membebani*) or possibly creating discomfort (*tidak enak*, *sungkan, rikuh*). Health professionals might notice this implicit wish, for instance, if the patient is in hospital and their appetite changes. One of the doctors in our study, for instance, said that sometimes the patient eats better when a specific family member is present during hospitalization. Therefore, presence of family members in daily caring activities is an important expectation that sometimes remains implicit.

#### Taking the Initiative in Providing Financial Support

Providing financial support was an expectation that should be fulfilled, particularly by family members who are not directly caring for the patient. Providing financial support can therefore be seen as a form of partial substitution for a physical presence in caring. Since asking for financial support is considered sensitive and might be inappropriate to discuss openly, it mostly remained an implicit expectation. Family members are, therefore, expected to take the initiative to provide financial support without being asked.

Discomfort may come from expressing personal or private individual needs, such as the need for diapers. By taking the initiative to provide diapers for instance, family members could reduce the discomfort in explicitly discussing personal needs. Sri, a daughter of a bedridden patient with unoperated hip dislocation, told us about the support her family received from other family members:

*Sri: Her grandchild took the initiative to buy diapers for her. We do this together. Her children living in other towns and other countries also help, sending some money. Everybody thinks together (regarding how to care for her). (FP06).*

The ethnographer also observed a portable toilet device in the patient’s bedroom, which has been purchased by the patient’s daughter living overseas, possibly earning a higher income than Sri.

Mawar, the patient with breast cancer referred to above, spoke about the difference between her daughter and her son. Her daughter took the initiative to offer financial support when she needed to travel to the hospital by asking, “*Mom, do you have some money to buy gas? Here is some pocket money for you, to buy gas or snacks*.” Her son never showed this kind of initiative, which made Mawar sad.

Sugeng, a patient who had had a stroke and with a diabetic ulcer, had a more explicit approach, as he did not refrain from asking for money from his family members, neighbors, and colleagues. He said, jokingly, “*aku njaluk duite*” (a Javanese sentence meaning “I ask for your money”) to his old school friends. Sugeng admitted that he was not shy anymore in asking for help and openly admitted that he received donations and support. Sometimes, Sugeng also used social media to show his needs, such as for internet bundles, which were then followed by his friends sending him some money or buying him some food. The example of Sugeng’s explicit communication shows a deviation from the usual norm of implicit expectation. This financial support, explicitly requested, was an important form of assistance. However, because it was more explicitly requested, it could not be ensured that it comes from a sincere attention to care for the patient.

### What makes implicitness a value?

Expectations, wishes, or desires could not usually be simply made explicit due to the importance of the value of implicitness in family care. Implicitness is a value because it creates a way to ensure sincerity and maintain harmony. When a family member fails to act upon this value, they may be perceived by the patient, other family members, or health professionals as bad caregivers.

#### Ensuring Sincerity

The value of implicitness aims to ensure sincerity by providing a way for people to take initiative in providing care, without being asked. We learned that keeping expectations implicit and not transforming them into requests or demands could be a test of sincerity. When family members accept an explicit request for help, it is difficult to know whether the help comes sincerely or not. There is a possibility that family members do it to avoid the discomfort of declining the request and to remain socially acceptable. On the contrary, if family members do something and contribute to care without explicit request, it is considered that their help truly comes from the heart. By not explicitly saying ‘what I need’ and ‘what to do’, the action taken by family members is felt to be genuine and sincere. When an act of care is done sincerely in this way, the care receiver will feel less like a burden. The importance of sincere affection is represented in Desi’s story, in which she emphasized the concept of *gemati* (a Javanese word meaning loving and caring sincerely (Supriyadi 2024). Desi is an elderly woman who took care of her adult bedridden son, who had suffered a severe stroke. The patient’s son, a young adult of around 20 years old, needed to be instructed by Desi to care for the patient:

*Desi: I told him (the patient's son/Desi's grandson), “Whether you want to wash him or not, it's up to you! If I don't tell you, he won't get washed. How is this possible?”. I feel a little upset with him, why isn't he* gemati* at all?*

*Interviewer: What is* gemati*?*

*Desi: Love. He does not truly love his father, maybe only a glimpse of it. (F05)*

She argued that when a person is *gemati,* he or she should not need any nudging or instruction to take care of loved ones. Desi told us that her grandson was not quick enough to understand the needs of his father, because he lacked sincerity. She preferred not to ask explicitly, because it would invalidate the sincerity of the action.

#### Maintaining Harmony

Implicitness also aims to maintain harmony by preventing arguments or conflicts and discomfort that can arise from discussing expectations explicitly. From previous cases, such as the quote from Sri, we learned that implicit expectations, such as expecting family members to buy diapers or to send money for the primary family caregivers without being asked, could avoid possible discomfort related to being vulnerable. The absence of discomfort may be considered to be a part of a state of harmony. Meanwhile, Dahlia’s case below provides an example of how explicit communication can lead to conflict and confrontation. Dahlia took care of her divorced father, who now had daughters from his new wife:

*When Dahlia’s father was hospitalized in another city, she told her half-siblings that she and her husband had to go back and forth, perhaps almost 1600 km over the course of ten days of hospitalization. Her half-siblings openly accused her of making things up. “Is father really sick? Is this your agenda to ask for our money?” (FW16)*

Although this is an extreme example, Dahlia’s case points out that explicitness may trigger conflict and uneasiness among family members. Dahlia and her husband were offended when they heard her half-siblings’ reaction. They hoped for some empathetic response such as “*Thank you for accompanying our father*”. The financial support, although it is also expected, was something they did not ask for. For Dahlia and her husband, it would have been better for Dahlia’s half-siblings to stay silent rather than to accuse them of making up stories.

Another example of implicitness being used to sustain harmony was shown by Nardi, a patient with paraplegia and a pressure ulcer, who refused assistance from the social worker to help solve family financial problems. He told us, “*I did not want to make a fuss in the family*.”

The above cases reveal the strong ideals and norms of sustaining harmony within the family, and avoiding arguments or conflict; maintaining harmony through keeping certain issues implicit is better appreciated, even if it sometimes means bad care for the patients.

### The Ethical Implications of Being Implicit about Expectations

It is clear from our findings that implicitness plays a significant role in everyday palliative care situations within families. Implicitness as a value comes with good intentions and is seen or used as a form of care; however, it does not always result in positive outcomes. Implicitness by itself has the potential to lead to underlying tensions that are not addressed properly. These challenges could arise in the daily practices of family care.

When expectations remain implicit, it is possible that other family members cannot instinctively grasp the needs of care recipients or family caregivers. They do love and are concerned for the patient, but they genuinely do not know how to start helping. The patient and primary family caregiver refrain from asking for help due to the idea of maintaining sincerity and preserving harmony. This reflects a problematic double-bind emotional dilemma, first described by Bateson et al. (1956), leading to unmet needs for support. While making things explicit might not be good since it spoils sincerity and harmony, remaining implicit may also not be good and could lead to inadequate support or underlying and unresolved conflict.

It is also possible, however, that family members lack the sincere and attentive characteristics needed to provide care for a patient. After several unsuccessful attempts to ask for help explicitly, the patient or their main caregiver resorts to implicit communications, refraining from further request for help and keeping their struggles to themselves. This situation frequently occurred in response to relatively young male family members’ lack of care. Mawar, the patient with breast cancer, told us that she once explicitly asked her son for help with some money to go to the hospital. Her son did not respond at all. She felt deeply saddened by the lack of support from her son, and so decided that she would not ask him for any help again so as not to be disappointed. She still hoped for her son to be more attentive, but she could not complain because it would feel like she was giving instructions:

*Mawar: I feel uncomfortable if I have to give “instruction”, for me it is not appropriate if the parents say these things (how to better care) to the children. If I need anything, I will wait until I or my husband can get it ourselves (P10-2).*

Another example of unresolved tension due to implicitness and resulting unmet expectations can be found in Putra and Putri’s family who were caring for their elderly, bedridden mother with severe osteoarthritis and multiple morbidities. This is a lower-middle income family living in a rural area of the hills:

*Interviewer: Have you ever told your other family about your needs?*

*Putra: No. Our principle is “not asking (for help) from anyone unless they give it sympathetically.” Even if I ask (for help) from my wife's older sister, I forbid her. Don’t ask. We must try ourselves.*

*Putri: Oh God, (actually) it would be nice if my sister asked “How is mother doing? What food does she eat? This is some money to help.” If that was the case, that would be very appropriate. (F07)*

The quote shows the unmet implicit expectations of how family members should provide care despite not living together, such as showing attention by asking about the patient’s condition. The tone of Putri's sentence shows resentment toward her sister. It shows unresolved tensions between family caregivers living together with the patient and other family members living elsewhere. Moreover, this case emphasized how implicitness shaped the expectation of financial support, as illustrated by Putri, who expressed her expectation of her sibling to say, “*This is some money to help*.”

Melati’s story reveals how unresolved tensions could also occur between close family caregivers in the home. Melati is a mother of an adult child with cerebral palsy and severe ear infection. Although her husband cooperated very well in doing physical caregiving activities, Melati also expected him to provide emotional support:

*Melati: I have never been able to talk and discuss things with my husband [...] Sometimes, it feels like two foreigners living together. When I tried to share my burden, he said: “You should not complain like that, this daughter has been given to us by God Almighty for a reason.” I know that is true, but I just want to tell my story. (F08)*

This quote shows a very complex interplay between the different expectations of Melati and her husband. Expectations are not static and may shift, as illustrated by Melati who wanted to talk about difficulties, while her husband wanted to not complain about their difficulties. It could also show that Melati favors less implicit communication than her husband. These differences are not resolved and lead to dissatisfaction. By asking Melati not to talk about her burdens, Melati’s husband dismissed her emotional needs and undermined her struggle. The tension between them was also rooted in wider gender norms, extending to different opinions regarding Melati’s activity with her friends, her idea to return to college, and her parenting style. However, these tensions were not sorted out, due to Melati’s perception that a wife’s responsibility includes “*following husband’s every word*.” Therefore, her expectations for her husband to provide more sincere affection were not explicitly discussed and not resolved.

### Strategies to Resolve the Problems of Implicitness

The ethnographic data revealed three common strategies among participants to address the problems arising from implicitness of expectations: for families to accept difficult circumstances, for families to share their difficulties with health professionals, and for health professionals to take the initiative in exploring further and acting sensitively.

Firstly, some caregivers, like Dahlia, chose to accept the circumstances and come to terms with some of their unmet desires:

*Dahlia: Actually, I am physically tired. I want someone to take over (this caregiving role), but my siblings live far away, what can I do? I felt upset with them. Because I am here alone, no siblings live here in this town, so I have to accept that I do everything by myself. (FP01)*

Since Dahlia’s siblings live in other cities, she had to come to terms with this circumstance and rely on her husband and children to support her physical caregiving duties. Fortunately, her siblings still could provide financial support by regularly sending money to help with caregiving costs.

Secondly, some families chose to share their problems with health professionals. Health professionals often become a venting outlet for patients or the primary family caregivers. As an external party, health professionals are sometimes considered a safe space to express more explicitly their expectations and concerns. When communicating among family members themselves, patients and families may fear adding more burdens or creating conflict. Health professionals, however, could have or hear conversations about these expectations, especially during prolonged exposure to the patient, such as during home care visits. For instance, family caregivers told Fony, their primary care physician, that they often felt unappreciated for their caregiving, while the patient only wanted to see other family members who are not there on a daily basis:

*Fony: The primary family caregivers who are tired from taking care of the patient from A to Z, they are always blamed for everything. Meanwhile, the patient waits for visits from other family members, other children, who live far away. The primary family caregivers are more tired than those distant family members who only send money. I don’t know what to say. (FGD 2)*

Nurses, particularly those who are involved in homecare services, can delve deeper into family conditions, observe family dynamics, and listen to the whole story. Yusuf, a homecare nurse, shared his experience of hearing different family members express dissatisfaction with each other's caregiving roles:

*Yusuf: We never limit the duration of our homecare visits, to ensure care needs are fulfilled. For example, a female patient and her son (A) talk about another son (B) not providing enough money. The next visit, it's the other way around, with son (B) telling stories about his brother (A) not helping with hospital appointments. This happens frequently. (FGD 1)*

Third, some health professionals tried to explore the situation further when noticing a lack of family presence and involvement in patient care. Some patients, due to the feeling of discomfort and the fear of becoming a burden, cannot explicitly express their wish to have their family members by their side and use “cryptic language codes” that require sensitivity from health professionals to fully understand what they mean. Dahlia and her husband told us they feel it might be inappropriate to share family problems with health professionals but would appreciate it if health professionals took a proactive approach in exploring these issues first.

Mala, a palliative care physician, shared her communication techniques with other health professionals, noting the importance of validating the concerns of family caregivers and offering to help facilitate communication:

*Mala: When we hear patients’ and families’ stories, perhaps we feel confused and sad, but we can try to validate the feelings: “I understand what you feel right now, it must be difficult for you and the family. Let’s try to find the solution together. What do you expect from other family members? If you can’t say it directly, please feel free to tell it to us, perhaps we can be a bridge to communicate this with other family members”. (FGD 1)*

Mala expressed that she herself is still learning how to communicate effectively, since every word choice she uses will influence the psychological well-being of the patient and family. To improve her communication skills and provide more holistic care, she emphasized the need to practice more frequently. One good example is Astrid, a *Puskesmas* nurse, who visited a patient’s home to uncover the patient's true wishes and the real challenge within her family.

*Astrid: There was one patient who did not attend her medical appointments. She told us she was busy selling snacks at the local market every morning. When we visited her home, she looked so happy and excited. It turned out she wasn't selling snacks at the market but was actually staying at home. She had an adult son who was also at home and pretended to sleep when we arrived. We realized that she wanted to come to the* Puskesmas*, but she couldn't ask her son to take her there, so she came up with the “market” excuse. (FGD 2)*

Astrid’s visit turned out to be an important moment for the family. Although she did not directly communicate with the son about caregiving, she explained and emphasized to the patient the importance of routine check-ups in a way that meant the son could also hear this too. Her visit eventually inspired the patient’s son to improve as a caregiver. In the next home visit, Astrid learned that the patient was routinely attending the hospital appointments accompanied by her son. Another patient asked the *Puskesmas* doctor to emphasize to her, when her son was sitting next to her in the consultation, that “*You must come routinely for medical check-ups*”, hoping that the doctor's advice would motivate the son to help more without directly telling him what to do.

Health professionals also often felt less able to help after exploring patients’ and families’ difficulties, particularly when problems were related to the patient’s economic circumstances. One homecare nurse said, *“Of course I am confused. If the conflict is finance-related, what can we do anyway?”*. The head of a *Puskesmas* expressed, “*It is our duty to care, but if we have to think about their economic condition, we give up, this is the scope of someone else.*” One possibility to solve this problem according to the focus group discussion in our study would be to connect the patient and the family caregivers with available resources, such as institutional and regional social funds, or external philanthropy organizations.

## Discussion

**Fig. 1:**
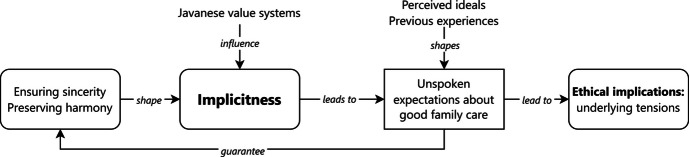
Conceptual map of the value of implicitness

From the ethnographic fieldwork, we illustrate how the value of implicitness shapes everyday family care, particularly for patients in need of palliative care. We were intrigued by the fact that implicitness strongly influences everyday palliative care practices, despite the ongoing modernization of Javanese society and shifts in family structures. We found that patients and family caregivers have different levels of implicitness in communicating their expectations. The common expectations hidden by this value are that families should be sincerely involved in daily caring activities and take the initiative in providing financial support. We argue that implicitness is an important value for people because it helps to maintain a certain type of family harmony and sincerity with caregiving. However, this value could also lead to underlying and unresolved tensions that affect the well-being of patients and family members. Some family members may not live up to implicit expectations, but it is also complicated for patients and primary family caregivers to express expectations directly because it may jeopardize the sincerity of actions or disrupt family harmony. Figure 1 provides a conceptual map of the value of implicitness.

We argue that the value of implicitness is relevant particularly for palliative care provision in countries with higher contextual communication, as implicitness is likely to be more prevalent and so more likely to shape the well-being of both patients and their families who are dealing with life-limiting illnesses. Patients and their families are crucial, inseparable targets of palliative care. The holistic approach to palliative care, which also considers the social and psychological aspects of both patients and families, is essential for those facing all types of life-threatening illnesses (World Health Organization 2021). It is crucial to provide good care for them, as they are facing multifaceted, serious health-related suffering (Doubova et al. 2023; Knaul et al. 2018).

Our findings are similar to previous studies by Ahlin and Sen (2020); Cagle and Munn (2012) and Tian et al. (2023), which illustrate the expectations to be hands-on and to provide practical support are part of the typical obligations of primary family caregivers. Meanwhile, the ability to provide financial support from a distance serves as an alternative for long-distance family members to fulfill their duty. As described by Neller et al. (2024), the caregiving role stems from personal, cultural, and societal expectations. Family involvement in care is nothing new but is becoming more common and challenging, especially with more complex medical treatments (Schulz et al. 2020). To provide better care for the whole family in a situation of palliative care, we need to assess how families communicate (Sherman 2019) and, in this case, whether the value of implicitness shapes how they convey their care expectations and affects the patient and the family. Other studies have described implicitness in terms of collusion and euphemisms around diagnosis and prognosis among Asian palliative patients (Chaturvedi et al. 2009; Martina et al. 2022). This study, however, differs from previous studies as we specifically elaborate on the influence of the value of implicitness in everyday family involvement in palliative care settings.

We found that the implicit expectation to provide physical caregiving is often directed at family members living in the same household as the patient, mostly female. This is a common worldwide phenomenon, as deep-rooted gender norms often assign caregiving responsibilities to women (Stall et al. 2023; Xiong et al. 2020). However, a recent study revealed that male caregivers tend to spend a similar amount of time in caregiving, even though female caregivers devote more hours to household chores (Pacheco Barzallo et al. 2024). This illustrates that the gender gap in caregiving is narrowing, which might explain our findings that female caregivers also expect other family members (e.g. male caregivers, distant relatives) to take a more active role in providing physical care.

We also found a family with higher income and education level who tend to incorporate less implicitness. Although it is one phenomenon, we might be able to understand it by reflecting on previous studies. Gao et al. (2022) found that socioeconomic status (monthly income and education levels) has a significant direct effect on the parent–child relationship. According to Chen and Berdan (2006), parents with higher education levels and professional occupations are more responsive to the negative behaviors of their adolescent children, which helps them better regulate interpersonal relationships and conflicts. These findings might explain why families with higher socioeconomic status tend to engage in more open conversations and rely on less implicitness in their dynamics.

We argue that implicitness is a value since it underpins notions of sincerity and harmony. We found that the provision of sincere attention and affection by family members were considered important aspects of good care. The concept of sincerity may stem from the Javanese-Islamic value of *ikhlas,* which emphasizes the importance of not expecting something in return for providing care (Wicaksono et al. 2024a). Implicitness also reflects the concepts of tacit expectations and tacit knowledge. It is implicitly expected for family members to have the tacit knowledge of how to best provide care for patients. Tacit knowledge itself is a knowledge that is difficult to make explicit, and often done automatically (Hager 2012). In a previous Dutch study, parents’ tacit knowledge for understanding subtle signs of patients with intellectual and multiple disabilities contributed to good care. For instance, family members were able to have the instinct to precisely predict the patient’s wishes and act upon these (Kruithof et al. 2024). While tacit knowledge emphasizes cognitive ability, the sincerity we found in our study extends from the affection that drives the cognitive and psychomotor ability to finally perform the caregiving instinctively.

Additionally, implicitness helps to maintain harmony, making it an important value in family care. By staying implicit and not turning expectations into demands, people are trying to maintain social harmony. The phenomenon of keeping things implicit and unspoken reflects the Javanese attitude towards preventing controversy and conflict in the public spotlight (Kawamura 2011), which further strengthens the importance of harmony. The communication pattern between family members in this study mostly preserves family conformity and harmony while having limited in-depth conversation about how to provide good care. This is particularly the case for families living in more rural areas with lower socio-economic status. Koerner and Fitzpatrick (2006) categorized this type of family as *protective* families, in which conflicts are perceived negatively, and communication skills are not much practiced. Families with higher conformity tend to avoid conflict, as they have lower self-concern and assertiveness (Shearman and Dumlao 2008). This might explain why most of our participants value implicitness in caregiving, as it reflects a lower self-focus and a greater desire to avoid conflict and maintain harmony. Nevertheless, one participant with complicated family dynamics experienced conflict when discussing caregiving explicitly with distant family members. Caregiving-related conflicts are commonly experienced by family caregivers and are associated with caregiving burden, as shown by Dieker et al. (2024). This conflict might also happen because preexisting family circumstances, or because some information was not sensitively discussed between the family members.

Case et al. (2005) have described how people avoid certain information when it might induce discomfort or dissonance. Family members learned overtime to understand what issues might be sensitive to discuss, therefore, they know how to behave in sensitive situations and act according to the implicit rules within the family (Crane et al. 2020). Hence, living up to implicit expectations can have positive implications for care activities and family relationships in general.

The implicitness found in our study may reflect the concept of silence. Silence is a response to the demands of everyday life, where staying silent and not showing suffering is regarded as a form of moral and social care by avoiding negative social relations (Dragojlovic and Samuels 2021). Silence and implicitness in our findings can be interpreted as a wish not to make caregiving more difficult. When caregiving is already a difficult activity, staying silent and not explicitly expressing needs can be a form of preventing difficulties from becoming more prominent. Silence may not be a sign of the absence of agency or autonomy, but instead a well-rehearsed refusal to speak out loud (Dragojlovic and Samuels 2023). Therefore, ensuring expectations remain implicit can also be seen a form of care.

However, despite intentions to ensure sincerity and maintain harmony, tensions and conflicts can often still occur beneath the surface and remain unaddressed, creating a sense of pseudo-harmony (Lestari et al. 2013). When people find out that the outcome did not live up to prior expectations, they might experience disappointment which is often associated with feelings of anger. Anger is particularly experienced when another person is deemed responsible for the disappointing event (Zeelenberg et al. 2000). The avoidance of discussing caregiving topics, shaped by the value of implicitness, might therefore lead to anxiety and lower quality of life for caregivers (see also Wittenberg et al. 2021).

## Implications for Practice

It would be helpful for health professionals, particularly those from communities with a high contextual communication culture, to reflect further on how they can respond to the value of implicitness shaping family involvement in caregiving for palliative patients. One of the reflective ethical questions might be “*Given the problems arising from the value of implicitness, should health professionals make family expectations of caregiving more explicit?*” To answer this question, there is no simple one-size-fits-all solution. Deliberation on a case-by-case basis might be needed when deciding how to solve the problem (Bartholdson et al. 2022). We suggest, on the basis of our findings, that health professionals could start by first becoming more sensitive in acknowledging and recognizing this value from a cultural perspective. It is important to systematically explore values, such as implicitness, and integrate them into decision-making (Kelly et al. 2015). Therefore, health professionals could identify how implicitness can work well and how it can also pose problems in different circumstances. These problems might be aggravated when patients and primary family caregivers are not accustomed to or do not feel comfortable explicitly conveying their opinions, perspectives, needs, and wishes.

The health professional participants showed a willingness to further explore patients’ and primary family caregivers’ wishes, preferences, and challenges regarding how to provide care. This explorative activity requires sensitivity in identifying potential problems during initial contact with the patient needing palliative care. It is also helpful to have effective communication skills in palliative care, particularly to not only pay attention to medical issues but also to the personal challenges of patients and families (Engel et al. 2023). Furthermore, health professionals need to invest their time in sitting together with the patient and the family to thoroughly understand their care needs and expectations. These time investments can be applied during homecare visits or during medical appointments in healthcare facilities. Once trust has been built and a relaxed atmosphere has been reached, health professionals could start an informal dialogue that may lead to exploring support needs.

Even when health professionals have limited time, they could improve patients’ and families’ well-being by adopting a sensitive attitude toward listening and picking up subtle signs during conversations with patients and families. Health professionals could then identify symbols and codes within the conversation and potential problems experienced by patients and families. Then, health professionals could offer support to facilitate communication between patients and families. If it is appropriate, they could help persuade family members to be more actively involved in caregiving by using indirect communication. This way, health professionals might help patients and families who are hesitant to communicate directly with each other due to the value of implicitness. When the challenges are beyond the scope of health professionals, such as economic issues, they could try to connect patients and families with other support organizations.

## Strengths and Limitations

This study is the first to critically analyze the value of implicitness and its role in shaping everyday family involvement in palliative care settings. Driven by the empirical ethics approach in analyzing the data, we strived to unravel the positive and negative implications of this value for palliative care practices from daily caregiving situations. Furthermore, our ethnographic methods helped us to capture hidden expectations by looking closely at care practices through prolonged observation and repeated in-depth interviews. The focus group discussions in our study also helped us to show how health professionals understand implicitness and deal with the issues it raises.

The study, however, cannot generalize that all Indonesians apply the value of implicitness, given its ethnographic nature and small sample. The depth of understanding from ethnographic methods, however, has helped us develop understanding of that value as it might apply in other settings. The researcher who conducted ethnographic fieldwork (RBW) is also a medical doctor. This dual positionality might also have influenced participant responses during in-depth interviews, home observations, and focus group discussions. Moreover, due to the low socio-economic status and education levels of most participants, there might have been some misunderstanding regarding the role of the researcher. Some participants might have thought that the researcher could provide help for them, including financial support. Therefore, the researcher strove to clarify any misunderstanding during data collection.

## Conclusions

This paper sheds light on the role of implicitness as an important value relating to family involvement in palliative care settings, particularly in places with higher levels of contextual communication such as in Java, Indonesia. Health professionals should engage with the value of implicitness in palliative care situations, as this value can shape expectations in caregiving and lead to underlying tensions. Instead of pursuing Western ideas of “*making things explicit*” and assertive communication, health professionals should be able to sensitively recognize the importance of implicitness for patients and families, such as preserving sincerity and maintaining harmony. Therefore, cultivating a sensitive attitude to listen and pick up subtle signs during conversations with patients and their families could help uncover implicit expectations and their related problems. Health professionals could offer to facilitate communication among family members with different strategies, depending on the family circumstances. In this way health professionals may better enhance patients’ and families’ well-being based on the deeply rooted implicitness in Javanese community, without forcing every expectation to be explicitly articulated. An example of a sensitive strategy is the use of indirect orders. This involves implicit communication through intermediaries, such as when a nurse expects the family member to be more involved in caregiving at home but communicates this sensitively to the patient instead of the family member who is also present during that moment. However, it is also important to acknowledge that the value of implicitness might not work for everyone, and then health professionals could make sensitive decisions to intervene further and start a more open and assertive conversation among family members.

An example of a sensitive strategy is the use of indirect persuasion. This involves implicit communication through intermediaries, such as when a nurse speaks to a patient with the intention of persuading a family member.

## Data Availability

The datasets used and/or analyzed during the current study are not publicly accessible due to ethical and privacy concerns surrounding the sensitive nature of the material. However, they are available from the corresponding author (RBW) upon reasonable request.
